# Molluscicidal Activity of *Camellia Sinensis* (Green Tea) and *Camellia sinensis* var. Assamica (Purple Tea) Extracts Against *Biomphalaria pfeifferi,* the Major Vector Snail of Human Schistosomiasis in Sub‐Saharan Africa

**DOI:** 10.1155/japr/9932058

**Published:** 2026-01-02

**Authors:** Nickson Samoo, Ruth Nyangacha, Amos Mbugua, Ibrahim Mwangi, Charles K. Syengo, Martina Laidemitt, Martin Mutuku

**Affiliations:** ^1^ Department of Medical Laboratory Science, Jomo Kenyatta University of Agriculture and Technology, Nairobi, Kenya, jkuat.ac.ke; ^2^ Centre for Biotechnology Research and Development, Kenya Medical Research Institute, Nairobi, Kenya, kemri.org; ^3^ KEMRI Graduate School, Kenya Medical Research Institute, Nairobi, Kenya, kemri.org; ^4^ Centre for Traditional Medicine and Drug Research, Kenya Medical Research Institute, Nairobi, Kenya, kemri.org; ^5^ Department of Biology, Centre for Evolutionary and Theoretical Immunology, University of New Mexico, Albuquerque, New Mexico, USA, unm.edu

**Keywords:** *Biomphalaria pfeifferi*, *Camellia sinensis*, schistosomiasis

## Abstract

Freshwater pulmonate snails are prevalent across Kenya and act as intermediate hosts for trematode parasites, some of which are snail vectors for human schistosomiasis. Chemical molluscicides have rarely been used routinely in Kenya to control snails due to high costs of manufacture and the subsequent environmental concerns associated with their use. This study tested extracts from green tea and purple tea plants, *Camellia sinensis*, which are widely grown in Kenyan highland areas, against *Biomphalaria pfeifferi,* the obligate intermediate host for *Schistosoma mansoni*. Snails were exposed to five different concentrations of tea extracts (10, 20, 50, 100, and 150 ppm). All quantitative data analyses were done in R Version 4.4.0. Analysis by LCMS showed that the compounds present in the extracts were epicatechin, epigallocatechin, caffeine (the highest concentration of the compounds), theobromine, and assamsaponin C. The compounds identified by GCMS were hexadecanoic acid, caffeine, octadecenoic acid‐methyl, and octadec‐1‐ene. The crude extracts from the Kenyan tea plant *Camellia sinensis* (both green and purple tea) induced mortality of the *Biomphalaria pfeifferi*. Therefore, they can be explored as alternative plant‐based molluscicides against the vector snails of *Schistosoma mansoni*.

## 1. Introduction

Human schistosomiasis is a neglected tropical disease (NTD) that has coevolved with humans for centuries [1]. According to the World Health Organization (WHO) report, approximately 254 million people require treatment [2]. The largest portion of infection worldwide (90%) is in Africa [3]. Modern‐day schistosomiasis control programs in Africa primarily rely on chemotherapy using praziquantel (PZQ). Although this strategy is effective in reducing the prevalence and morbidity associated with the disease, it is not sustainable because reinfections do rapidly occur after successfully treating infected individuals [4, 5]. To sustain the gains made through drug‐based control strategies, there is a need to explore ways to incorporate snail control strategies that offer efficacy but are also relatively friendly to the environment for control and elimination of schistosomiasis and other snail‐borne diseases.

In the 1950s and 1970s, chemical mollusciciding was at the forefront of schistosomiasis control [6]. Compounds like copper sulfate, sodium pentachlorophenol (NaPCP), N‐trityl morpholine, and niclosamide were frequently used to control snails, particularly to control schistosomiasis in Asia, Africa, and South America [7]. It was not until the introduction of chemotherapy using PZQ in the 1970s that it displaced the use of molluscicides [6]. Although effective for snail control, synthetic chemical molluscicides are costly, unfriendly to the environment, and may have a long‐term negative impact on nontarget organisms like fish and amphibians in water [8]. Further, the biodegradation rate for synthetic compounds is incredibly slow, making them hazardous to the environment [9]. At present, there is limited use of chemical molluscicides because of their high cost, the risk of ecological damage, and the potential advancement of snail resistance to chemical molluscicides.

As an alternative to chemical molluscicides, plant‐based metabolites have been suggested to offer similar efficacy while reducing harm to the environment [10]. The use of plant‐derived molluscicides offers a simple and cost‐effective alternative to chemical molluscicides and could play a significant role in sustaining efforts to control and eliminate schistosomiasis in endemic areas. Several experimental studies have been conducted to assess plant species for their molluscicidal properties [11–13]. Also, different plant species have been identified as environmentally friendly alternative molluscicides [13–18]. Plant‐derived molluscicides meet the criteria for efficacy and safety to the environment and nontarget organisms and can be used to complement chemotherapy [19]. Alkaloids, saponins, cardiac glycosides, and therapeutic oils/terpenes are examples of metabolites that have demonstrated effectiveness in controlling snail populations [20]. Plant‐derived metabolites are beneficial to both the plants from which they are derived. These secondary metabolites act synergistically and confer protective advantages to plants, which include antifeedant properties, seed dispersal in animals, and attracting pollinators, among others [21]. Further, the synergistic properties of these plant phytochemicals make them effective for use in various industrial applications as well [22]. Natural plant compounds are easily accessible, inexpensive, environmentally friendly, and quickly biodegrade. There are fewer chances that molluscs will be resistant to them [12]. They seem to be the most reliable methods for reducing snail populations.

One plant that has shown remarkable potential as a source of plant‐derived molluscicide is the tea plant *Camellia sinensis* (L.) O Kuntze. *Camellia sinensis* (L.) O Kuntze was first described as *Thea sinensis* by Linnaeus (Linnaeus, 1753). The tea plant is said to be originally from Yunnan in China and Assam in India. There are two major variants, the small leaf variant from the temperate regions of China and the large leaf variant from India. However, other variants that were hybrids of the two variants were present in many locations between China and India [23]. Going by the earliest classification of the tea plant, the smaller leaved variants were classified as *Camellia sinensis* (L.) O Kuntze, the Chinese type and *Camellia assamica* (Masters) was the Indian variant with larger leaves [24]. Later, due to the close resemblance in morphology and biochemical characteristics, *Thea* and *Camellia* were considered to be the same genus. Today, tea is referred to as *Camellia sinensis* (L.) O Kuntze overlooking variations between species.

Kenya is one of the countries where cultivation of tea is done on a large scale. Globally, tea‐producing countries include China, India, and Sri Lanka. African countries such as Rwanda, Malawi, Uganda, and Tanzania join Kenya as major tea producers [25]. Tea cultivation requires optimum conditions. For all the regions in the world that tea is cultivated, suitable optimum ecological conditions should be met for optimum yields [26]. Tea is grown in areas with an average temperature of 26°C [27]. In Kenya, volcanic soils are ideal for tea cultivation. Acidic soils with a pH range of 4.0–5.5 support the required nutrients. Cultivation of tea in Kenya is done in areas experiencing an average temperature of 18°C–25°C. The average annual rainfall is between 1200 and 1400 mL [28]. Kericho is among the tea‐growing areas in Kenya with an altitude ranging between 1500 and 2700 m above sea level [29].

In Kenya, the vector snails that harbour the schistosoma parasite (for both *Schistosoma mansoni* and *Schistosoma haematobium*) are from the genus Biomphalaria and Bulinus snails, respectively. The snail *Biomphalaria pfeifferi* Krauss which primarily transmits *Schistosoma mansoni* was first described after being recovered from Umgeni Valley in Natal, South Africa. The snail was first described in 1848 by Krauss [30]. *Biomphalaria pfeifferi* is found in major lakes and rivers in East Africa. Snail distribution depends on certain ecological and physicochemical parameters that include water depth, presence of clay soil, and average temperatures of 25°C. These factors support the abundance of *Biomphalaria pfeifferi* snails in East Africa. The presence of riverine vegetation is an important factor for attachment, oviposition, and feeding of *Biomphalaria pfeifferi* snails [31].

A study by Cho et al. [32] showed the *Camellia sinensis* seeds exhibit great toxicity against the golden apple snail *Pomacea canaliculata*, Lamarck. Also, a study by Jia et al. [33] tested 4% tea‐derived saponins (TDSs) which were effective against the snail *Oncomelania hupensis* quadrasi. The tea seed formulation is currently known as “Luo wei” and is used for focal mollusciciding. The 4% TDS product derived from the seeds of *Camellia sinensis* has been evaluated and found to be effective against *Oncomelania hupensis* quadrasi snails (the prosobranch snail species responsible for the transmission of *Schistosoma japonicum*) with molluscicidal activity comparable to that of niclosamide, a commercially available synthetic molluscicide originally developed by the German company Bayer [17, 34]. This saponin‐based product is now commercially available. This study sought to evaluate the molluscicidal activity and phytochemical profile of *Camellia sinensis* (green tea) and *Camellia sinensis* var. assamica (purple tea) extracts against *Biomphalaria pfeifferi*, the major vector snails of *Schistosoma mansoni* in Kenya.

## 2. Materials and Methods

### 2.1. Collection of *Camellia sinensis*

**Figure 1:**
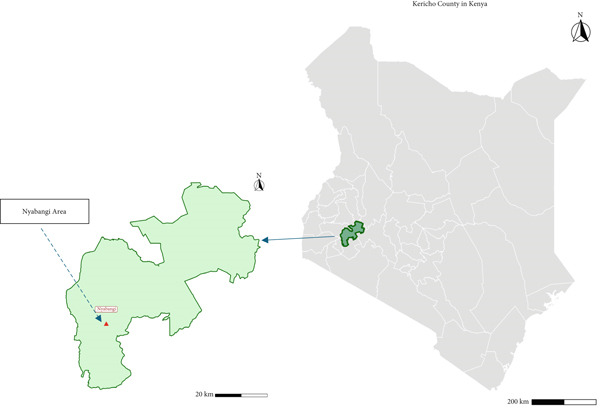
Map of Kericho county in Kenya. *Source:* Humanitarian Data Exchange (HDX) | Kenya IEBC Boundaries (2019).

The tea plants were collected in Kericho County, Nyabangi area (0°26 ^′^40.41348 ^″^S, 35°8 ^′^36.78396 ^″^E) (Figure 1). The plant parts (mature leaves, shoots, stembark, rootbark, and seeds) were transported in sisal bags to preserve them as they were transported to the Center for Traditional Medicine and Drug Research (CTMDR) at the Kenya Medical Research Institute (KEMRI) for processing.

The plant parts were harvested from mature tea plants with the help of a taxonomist who assisted in identifying the two varieties of *Camellia sinensis*. Voucher specimens were deposited at the University of Nairobi (UoN) Herbarium under the Voucher Number NS/UON/2023‐001. The different plant parts were air‐dried at room temperature under shade for 21 days and then pulverized using a laboratory mill (Christy & Norris Ltd., Chelmsford, England) at CTMDR‐KEMRI. The powders were packed in air‐tight polythene bags, labeled, and stored.

**Figure 2:**
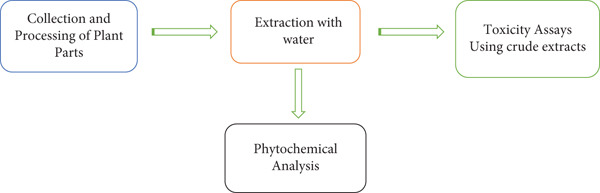
A diagrammatic representation of plant extraction, biochemical characterization process, and testing for molluscicidal activity.

The powdered plant extracts were soaked in water as shown in Figure 2. They were then heated in a water bath at 60°C for 1 h. After heating, the solution was filtered by passing through a cotton gauze. The filtered solution was then put in a glass flask and coated with dry ice and acetone. After which they were freeze‐dried, transferred to an air‐tight container, weighed, labeled, and stored at 4°C. Table 1 below shows the extracts with their respective yields.

**Table 1:** The plant samples collected and their respective yields in grams.

**Plant (*Camellia sinensis*)**	**Plant Extract**	**Weight (grams)**	**H**_**2**_ **0 volume (mL)**	**Yield (grams)**
Purple tea	Seeds	50	250	7
Green tea	Seeds	100	500	17
Green tea	Rootbark	62	250	6
Green tea	Stembark	100	500	7
Green tea	Young shoots	100	500	11
Green tea	Mature leaves	100	500	15

### 2.2. Liquid Chromatography

Samples were sonicated in pure methanol, filtered through 0.22‐*μ*m syringe filters, diluted in the mobile phase (methanol: H2O; 1:1) and transferred to LC autosampler vials for analysis. The instrument parameters included a Kinetex 2.6 *μ*m XB‐C 18 column (150 × 3 mm) paired with a Phenomenex Security‐Guard Ultra cartridge. The mobile phase consisted of 0.1% water with 0.1% formic acid and methanol. The gradient program started with 10% B for the first 0.5 min, increased to 50% B by 5 min, then to 90% B by 8 min, maintained 90% B until 11 min, and finally returned to 10% B from 11.01 to 20 min. The flow rate was set at 0.25 mL/min, with an oven temperature of 40°C and an injection volume of 5 *μ*L. The MS interface used electrospray ionization (ESI) in full scan mode with polarity switching, covering a range of 70–1900 m/z. Nitrogen gas flowed at 2.0 L/min for nebulizing and 15 L/min for drying, with MS temperatures set at 250°C for the desolvation line and 400°C for the heating block.

### 2.3. Gas Chromatography

After the samples were sonicated in pure methanol, filtered through 0.22‐*μ*m syringe filters, diluted in the mobile phase (methanol: H2O; 1:1), only 1 *μ*L of the sample was injected for analysis. Separation was conducted using the SH‐Rxi‐5Sil MS capillary GC column (30 m, 0.25 mm ID, 0.25‐*μ*m film thickness). Ultrapure helium served as the carrier gas at a flow rate of 1.0 ml/min. The injection temperature was maintained at 200°C, employing the split injection mode with a 10:1 ratio. The oven temperature program was as follows: initial temperature 50°C (held for 1 min); increased by 20°C/min to 200°C (held for 1 minute); and then raised by 5°C/min to 300°C (held for 30 min). Samples were analyzed in full scan mode ranging from 50 to 700 m/z. The ion source temperature was kept at 200°C and the interface temperature at 280°C.

### 2.4. Collection and Molluscicidal Testing of *Biomphalaria pfeifferi*

Target snails comprised members of the freshwater snails of medical and economic importance in Kenya, namely, *Biomphalaria pfeifferi.* The snails were obtained from Kisumu and maintained in the schistosomiasis laboratory, Centre for Biotechnology Research and Development (CBRD), Nairobi. Both juvenile and adult snails were tested. We collected *Biomphalaria Pfeifferi* in Asawo River (0°18 ^′^42.7 ^″^S, 34°55 ^′^00.9 ^″^E) in the month of July 2024, where we used the scooping method to get the snails attached to water plants along the river bank. The snails were then transported in open plastic dishes covered in some river vegetation to avoid desiccation and heat‐related stresses during transportation to the laboratory. We transported the snails to Nairobi, CBRD, where they were put into breeding trays and labeled appropriately. The snails were bred in a controlled environment with a light/dark cycle of 12:12. The room temperature ranged from 25°C to 28°C. The snails were fed on lettuce and the breeding trays were cleaned once every 2 weeks to avoid water being cloudy from food residues.

Juvenile snails (approximately 1–3 mm in diameter) and adult snails (approximately 4–7 mm in diameter) were exposed to five different concentrations (10, 20, 50, 100, and 150 ppm) of the water extracts. The study used F1 generation for the tests, both adults and juveniles. For each test extract, 20 snails were put in each dish containing the extracts. Additional 20 snails were put in the negative control containing dechlorinated water only. The study primarily studied the green tea seeds which was done using five technical replicates. Comparison was done for the green tea seed against the other plant parts as well. As such, 120 snails were used for each plant extract which implies that 100 snails used in the test extracts and additional 20 for the negative control. For green tea seeds, a total of 600 snails were used. Additionally, when comparing green tea seeds and the other five extracts, 600 snails were used for the experiments. In total, 1200 snails were utilized. The snails were put in plastic containers with a capacity of 500 mL of water for 24 h. The same procedure was done for adult snails (> 4 mm shell diameter).

Observations time points were 30 min, 1, 2, 3, 4, 24, and 48 h postexposure. After 24 h, the snails were rinsed in dechlorinated tap water, then transferred into clean dishes containing dechlorinated tap water and fed on lettuce. They were allowed to recover from molluscicidal effects for 24 h. Data record of 24‐ and 48‐h time intervals on snail′s movements, retraction or no retraction, response to the presence of food items (lettuce), bleeding, and the number dead or alive were recorded. The snails were also observed under the microscope to check heartbeat and ascertain snail death.

### 2.5. Statistical Analysis

All quantitative data analysis was done in R Version 4.4.0 [35]. Kruskal–Wallis test [36] will be used to test the difference in retraction across multiple groups viz. tea extracts and concentrations. The nonparametric Kruskal–Wallis test is a nonparametric counterpart of the one‐way ANOVA that sacrifices the precision of discriminating means for the discrimination of stochastic dominance (i.e., the probability that a randomly drawn observation from one group will be greater than a randomly drawn observation from another). However, the test does so regardless of the underlying distribution for the measures [37]. The Dunn′s test with a Bonferroni adjustment was used to make multiple pairwise comparisons in event the Kruskal–Wallis test was significant [38]. Kaplan–Meier survival analysis was performed to evaluate the survival rates of snails, and survival curves were compared using the log‐rank test. A *p* value of less than 0.05 was considered significant. Probit analysis was also done using Microsoft Excel 2016 to check the lethal concentration (LC‐50) of the extracts.

## 3. Results

### 3.1. Biotoxicity Assays

*Biomphalaria pfeifferi* snails were exposed to green tea seed extracts and observed at different time points of 30 min, 1, 2, 3, 4, and 24 h postexposure. The figure below shows the average snail mortality of snails exposed to green tea seed extracts.

**Figure 3:**
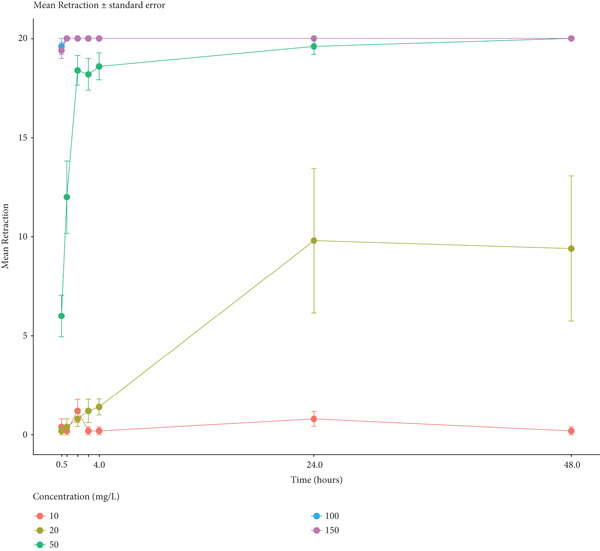
The average response of *Biomphalaria pfeifferi* to green tea seed extract.

From Figure 3, the plot above reveals that there is a concentration‐dependent effect. Lower concentrations (10 and 20 mg/L) resulted in minimal retraction over time. The medium concentration (50 mg/L) led to a clear time‐dependent increase in snail mortality. The highest concentrations (100 and 150 mg/L) induced an immediate, maximum retraction that was sustained throughout the time period. Further, there is a time‐dependent effect. The effect of the treatment developed over time, particularly for the 50 mg/L concentration, where snail retraction increased steadily from 1 to 4 h and reached its maximum by 24–48 h. Also, the effect of time differed by each concentration, suggesting a significant statistical interaction between concentration and time.

An overall Kruskal–Wallis test for group differences revealed that the median snail mortality did not differ significantly across the five groups (*p* = 0.7866). When comparing the group median differences at each concentration using the Kruskal–Wallis test, no significant differences were observed except at the 10 mg/L concentration (*p* = 0.01652 < 0.05). Further analysis of group median differences at 10 mg/L concentration using Dunn′s test revealed that Groups 2 and 5 significantly differed in terms of snail mortality. However, there were no significant differences between the rest of the groups (Table 2).

**Table 2:** Multiple pairwise comparisons of retractions across the five groups at 10 mg/L concentration.

**Group**	**Group**	**n**1	**n**2	**Estimate**	**Statistic (** **Z** **-score)**	**Adjusted** **p** **value**	**Significance**
5	4	7	7	−2.1	0.0327	0.3270	ns
5	1	7	7	−2.3	0.0217	0.2173	ns
5	3	7	7	−2.6	0.0103	0.1029	ns
5	2	7	7	−3.3	0.0010	0.0103	^∗^
4	1	7	7	−0.2	0.8734	1.0000	ns
4	3	7	7	−0.4	0.6669	1.0000	ns
4	2	7	7	−1.1	0.2511	1.0000	ns
1	3	7	7	−0.3	0.7864	1.0000	ns
1	2	7	7	−1.0	0.3231	1.0000	ns
3	2	7	7	−0.7	0.4732	1.0000	ns

Abbreviation: ns, not significant.

^∗^Significance

### 3.2. Comparison of the Different Plant Extracts Against *Biophalaria pfeifferi*

The snails were exposed to the different plant extracts that included mature leaf extract, green and purple tea seed extract, rootbark extract, young shoot extract, and stembark extracts. The average snail mortality for each extract for the different concentrations is shown in Table 3.

**Table 3:** Mean snail mortality of juvenile and adult *Biomphalaria pfeifferi* for green tea extracts at different concentrations.

**Snail stage/extract**	**Mean concentrations (mg/L) with standard deviation**						
**Juveniles**	**10**	**20**	**50**	**100**	**150**	**Mean mortality**	**(%mean mortality)**
Green tea mature leaf	0.00	0.00	0.00	0.29 (0.49)	0.29 (0.49)	0.11 (0.32)	1
Green tea seed extract	0.57 (0.83)	8.00 (6.53)	16.43 (6.52)	19.86 (0.84)	20.00 (0.00)	12.97 (8.89)	64
Green tea rootbark	0.43 (0.53)	0.29 (0.49)	15.43 (7.30)	19.86 (0.38)	20.00 (0.00)	11.20 (9.64)	56
Green tea shoot	0.00	0.00	0.29 (0.49)	1.43 (1.81)	2.57 (1.90)	0.86 (1.52)	5
Green tea stem	0.43 (1.13)	0.14 (0.38)	0.71 (0.76)	5.00 (7.16)	7.86 (8.82)	2.83 (5.74)	15
**Adults**							
Green tea seeds	1.14 (0.69)	1.86 (0.69)	16.43 (4.12)	20.00 (0.00)	20.00 (0.00)	11.89 (8.89)	60
Purple tea seeds	2.71 (2.43)	7.14 (6.64)	17.86 (4.06)	20.00 (0.00)	20.00 (0.00)	13.54 (8.08)	67

The results in Table 3 depict a trend of the different green tea extracts′ toxicity against *Biomphalaria pfeifferi.* The extracts could be ranked in terms of toxicity from the least toxic to the most toxic as follows: green tea leaves (1%), green tea shoot (5%), green tea stem bark (15%), green tea rootbark (56%), green tea seed (64%), and purple tea seed (67%).

The median retraction levels differed significantly across the five green seed extract concentrations (*p* < 0.001). Further analysis of differences among concentrations using Dunn′s test revealed that the 10 mg/L concentration significantly differed from 50, 100, and 150 mg/L, implying a higher toxicity of 50, 100, and 150 mg/L concentrations to eliminate the juvenile snails as compared to the 10 mg/L concentration. However, there were no significant differences between the concentrations of 50 and 100, 50 and 150, and 100 and 150 mg/L (Table 4).

**Table 4:** Multiple pairwise comparisons of the retractions among the five concentrations of green tea seed extract against juvenile *Biomphalaria pfeifferi* snails.

**Concentration 1**	**Concentration 2**	**n**1	**n**2	**Estimate**	**Statistic (** **Z** **-score)**	**Adjusted** **p** **value**	**Significance**
10	20	7	7	9.07	1.813676	0.697276	ns
10	50	7	7	15.8	3.156082	0.01599	^∗^
10	100	7	7	19.7	3.941532	0.00081	^∗^
10	150	7	7	21.1	4.227151	0.000237	^∗^
20	50	7	7	6.71	1.342406	1	ns
20	100	7	7	10.6	2.127856	0.33349	ns
20	150	7	7	12.1	2.413475	0.158012	ns
50	100	7	7	3.93	0.78545	1	ns
50	150	7	7	5.36	1.071069	1	ns
100	150	7	7	1.43	0.285618	1	ns

Abbreviation: ns, not significant.

^∗^Significance

As shown in Table 5, the five concentrations of purple tea seed extract led to significant median retraction levels of the adult snails (*p* < 0.001). Multiple pairwise comparisons of the five concentrations revealed significant differences between concentrations of 10 and 50 mg/L (*p* = 0.0221), 10 and 100 mg/L (*p* < 0.001), 10 and 150 mg/L (*p* < 0.001), 20 and 100 mg/L (*p* = 0.0293), and 20 and 150 mg/L (*p* = 0.0293).

**Table 5:** Multiple pairwise comparisons of the retractions among the five concentrations of purple tea seed extract on adults *Biomphalaria pfeifferi* snails.

**Concentration 1**	**Concentration 2**	**n**1	**n**2	**Estimate**	**Statistic (** **Z** **-score)**	**Adjusted** **p** **value**	**Significance**
10	20	7	7	5.286	1.054	1.0000	ns
10	50	7	7	15.357	3.061	0.0221	^∗^
10	100	7	7	20.214	4.029	0.0006	^∗^
10	150	7	7	20.214	4.029	0.0006	^∗^
20	50	7	7	10.071	2.007	0.4471	ns
20	100	7	7	14.929	2.975	0.0293	^∗^
20	150	7	7	14.929	2.975	0.0293	^∗^
50	100	7	7	4.857	0.968	1.0000	ns
50	150	7	7	4.857	0.968	1.0000	ns
100	150	7	7	0.000	0.000	1.0000	ns

Abbreviation: ns, not significant.

^∗^Significance

The five concentrations of green tea root bark extract led to significant median snail mortality of the juvenile snails (*p* < 0.001). Multiple pairwise comparisons of the five concentrations revealed significant differences between concentrations of 10 and 100 mg/L (*p* = 0.0065), 10 and 150 mg/L (*p* = 0.00253), 20 and 100 mg/L (*p* = 0.00298), and 20 and 150 mg/L (*p* = 0.0011). Hence a higher toxicity of 100 and 150 mg/L is observed to eliminate the juvenile snails as compared to the concentrations of 10 and 20 mg/L. However, the concentrations of 10 and 20 and 100 and 150 mg/L did not differ significantly (Table 6).

**Table 6:** Multiple pairwise comparisons of the retractions among the five concentrations of green tea root bark extract.

**Concentration 1**	**Concentration 2**	**n**1	**n**2	**Estimate**	**Statistic (** **Z** **-score)**	**Adjusted** **p** **value**	**Significance**
10	20	7	7	−1.071	−0.209	1.00000	ns
10	50	7	7	11.214	2.184	0.28949	ns
10	100	7	7	17.500	3.408	0.00653	^∗^
10	150	7	7	18.786	3.659	0.00253	^∗^
20	50	7	7	12.286	2.393	0.16718	ns
20	100	7	7	18.571	3.617	0.00298	^∗^
20	150	7	7	19.857	3.868	0.00110	^∗^
50	100	7	7	6.286	1.224	1.00000	ns
50	150	7	7	7.571	1.475	1.00000	ns
100	150	7	7	1.286	0.250	1.00000	ns

Abbreviation: ns, not significant.

^∗^Significance

In Table 7, the five green tea extracts (mature leaf, root bark, seed, shoot and stem) led to significantly different median retraction levels of the adult snails (*p* < 0.001). The leaf extract differed significantly from the seed and root bark extracts (*p* < 0.001), hence a higher toxicity of the seed and root bark extracts against the juvenile snails as compared to the leaf extract. However, the seed and root bark extracts did not differ significantly. The leaf extract did not differ from the shoot and stem extracts. The root bark extract differed significantly from extracts from the shoot and stem (*p* < 0.001 and 0.0054, respectively), implying a higher toxicity of root bark to juvenile snails as compared to the shoot and stem extracts. However, there was no significant difference between the shoot and stem extracts. The seed extract also differed significantly from the shoot and stem extracts (*p* < 0.001). Hence, a higher toxicity of the seed extract to the juvenile snails as compared to the shoot and stem extracts.

**Table 7:** Multiple pairwise comparisons of the retractions among the five extracts of green tea.

**Extract 1**	**Extract 2**	**n**1	**n**2	**Estimate**	**Statistic (** **Z** **-score)**	**Adjusted** **p** **value**	**Significance**
Leaf	Rootbark	35	35	61.886	5.578	0.0000	^∗^
Leaf	Seed	35	35	74.829	6.744	0.0000	^∗^
Leaf	Shoot	35	35	13.100	1.181	1.0000	ns
Leaf	Stem	35	35	23.471	2.116	0.3439	ns
Rootbark	Seed	35	35	12.943	1.167	1.0000	ns
Rootbark	Shoot	35	35	−48.786	−4.397	0.0001	^∗^
Rootbark	Stem	35	35	−38.414	−3.462	0.0054	^∗^
Seed	Shoot	35	35	−61.729	−5.564	0.0000	^∗^
Seed	Stem	35	35	−51.357	−4.629	0.00004	^∗^
Shoot	Stem	35	35	10.371	0.935	1.0000	ns

Abbreviation: ns, not significant.

^∗^Significance

### 3.3. Probit Analysis

Probit analysis of green tea seed, purple tea seed, and green tea root bark was done. These three extracts exhibited the most toxicity against both the juvenile and adult *Biomphalaria pfeifferi* snails.

The probit analysis showed that the LC‐50 of green tea seed is at 27 ppm (Table 8). This implies that the concentration of 27 ppm will lead to snail mortality of half of the *Biomphalaria pfeifferi* exposed to green tea seed extract.

**Table 8:** The lethal concentration 50 (LC‐50) of the green tea seed.

**Concentrations**	**Log 10 (Conc)**	**% dead**	**Probit**	
10	1	5	3.36	*y* = *a* *x* + *b*
20	1.301029996	40	4.75	*y* = 3.65*x* + (−0.232)
50	1.698970004	80	5.84	5 = 3.65*x* − 0.232
100	2	95	6.64	5.232 = 3.65*x*
150	2.176091259	100	8.09	*x* = ^`^1.433
Intercept	−0.231968151			LC50 = antilog x
*X* Variable 1	3.649646244			LC50 = 27.102

The probit analysis of purple tea seed implies that a concentration of 23 ppm is enough to cause snail mortality of half the population of *Biomphalaria pfeifferi* exposed to purple tea seed extract at any given time (Table 9).

**Table 9:** The lethal concentration 50 (LC‐50) of purple tea seeds.

**Concentrations**	**Log 10 (Conc)**	**% dead**	**Probit**	
10	1	12	3.77	*y* = *a* *x* + *b*
20	1.301029996	20	4.16	*y* = 4.2*x* + −0.75
50	1.698970004	88	6.18	5 = 4.2*x* − 0.75
100	2	100	8.09	5 = 3.45*x*
150	2.176091259	100	8.09	*x* = 1.37
Intercept	−0.753384562			LC50 = antilog x
*X* Variable 1	4.16542841			LC50 = 23.44

As shown in Table 10, approximately 39 ppm concentration of green tea rootbark is enough to cause the death of more than half of *Biomphalaria pfeifferi* snails.

**Table 10:** The lethal concentration 50 (LC‐50) of green tea rootbark.

**Concentrations**	**Log 10 (Conc)**	**% dead**	**Probit**	
10	1	1	2.67	*y* = *a* *x* + *b*
20	1.301029996	2	2.95	*y* = 4.7*x* + (−2.5)
50	1.698970004	75	5.67	5 = 4.7*x* − 2.5
100	2	95	6.64	7.5 = 4.7*x*
150	2.176091259	100	8.09	*x* = 1.596
Intercept	−2.544810283			LC50 = antilog x
*X* Variable 1	4.73870095			LC50 = 39.45

### 3.4. Survival Analysis

Survival analysis was done, and Kaplan–Meier curves indicate the time taken by the snails to die. The curves in Figure 4 also indicate the time it took for half of the snails in the extract to retract before being confirmed as dead.

Figure 4The survival curves of snails exposed to the different tea extracts: (a) the survival probabilities for juvenile snails exposed to green tea seed extracts, (b) the survival probabilities of juvenile snails exposed to purple tea seed extract, and (c) the log‐rank *p* value of 0.04996578 indicates a significant result at the 5% level of significance. However, the snails recovered in the green tea shoot extracts as no extracts reduced the survival of the snails completely (d) The log‐rank *p* value of 0.182 indicates a nonsignificant result at the 5% level of significance. Hence, there is no survival difference between the five concentrations for green tea leaf extract: (e) the log‐rank *p* value of 0.23 indicates a nonsignificant result at the 5% level of significance. Hence, there is no survival difference between the five concentrations for green tea root bark extract: (f) the log‐rank *p* value of 0.17 indicates a non‐significant result at the 5% level of significance. There is no survival difference between the five concentrations for green tea stem extract.

**Figure:**
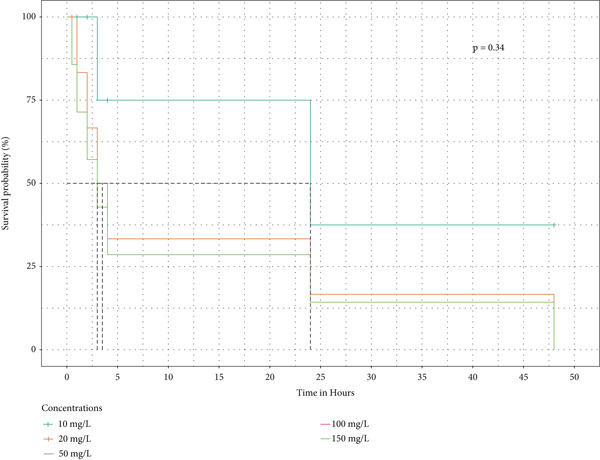
(a)

**Figure:**
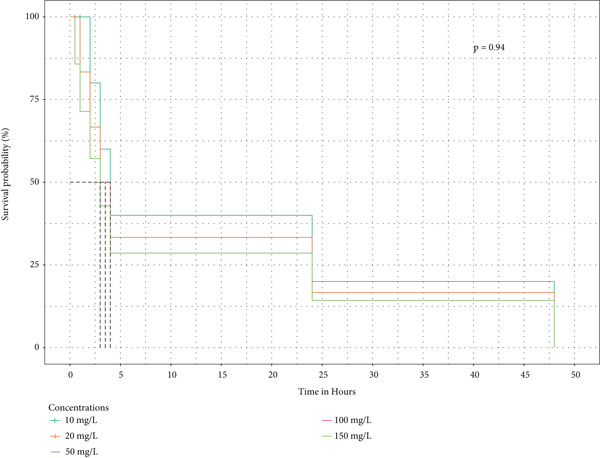
(b)

**Figure:**
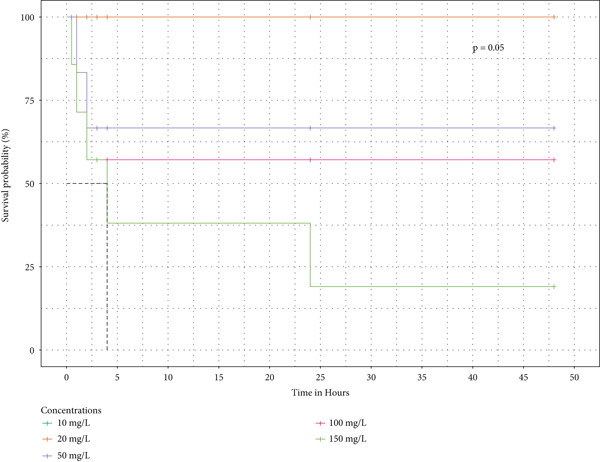
(c)

**Figure:**
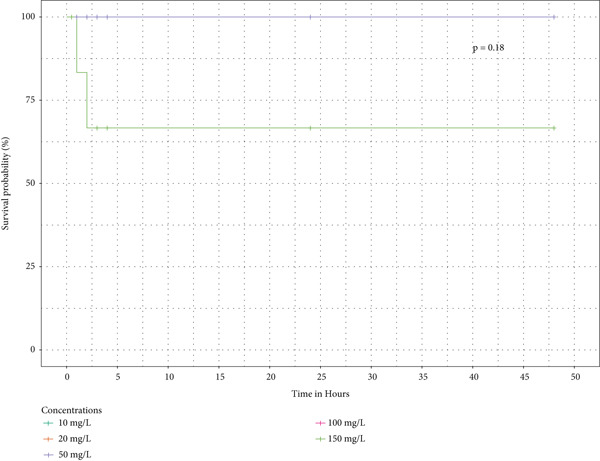
(d)

**Figure:**
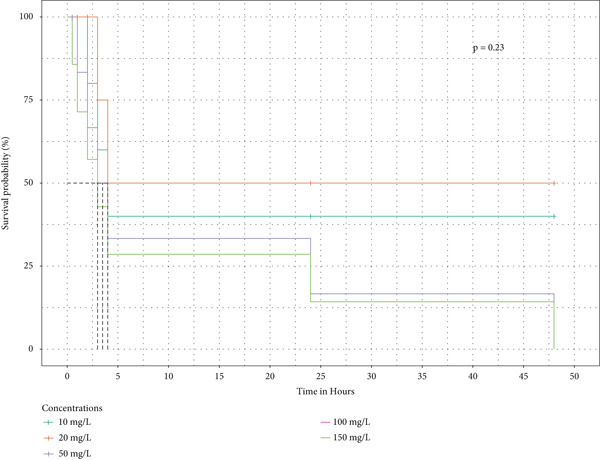
(e)

**Figure:**
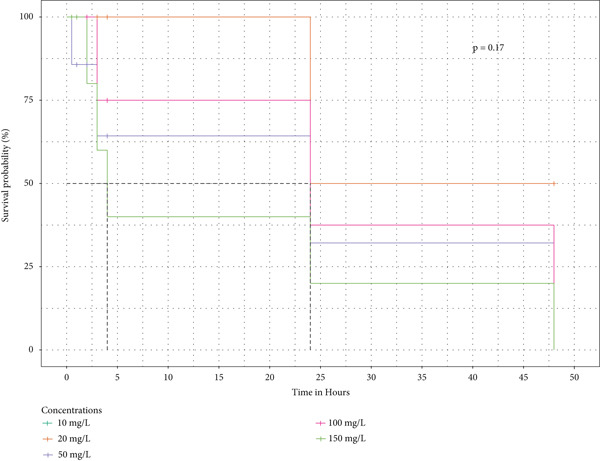
(f)

### 3.5. Liquid Chromatography

Liquid chromatography was done using a Shimadzu LCMS 8040 machine. Compounds were identified by observing dominant peaks and extracting the chromatograms as shown in Table 11.

**Table 11:** The table below shows the compounds identified by LCMS within the different plants.

**Plant extract**	**Compound identified**	**Retention time (Rt) (min)**	**Molecular weight (g/mol)**	**M + H**	**M + CH3OH + H**	**M + FA-H**	**Molecular formulae**
Green tea seeds	Assamsaponin C	12.214	1231.3	1232.31	1264.333489	1276.3	C_59_H_90_O_27_
Purple tea seeds	Assamsaponin C	12.18	1231.3	1232.31	1264.333489	1276.3	C_59_H_90_O_27_
Green tea mature leaf	^∗∗∗^	^∗∗∗^	^∗∗∗^	^∗∗∗^	^∗∗∗^	^∗∗∗^	^∗∗∗^

Green tea stem bark	Epichatechin	8.255	290.26	291.277	323.303489	335.268	C_15_H_14_O_6_
	Epichatechin gallate	8.695	442.37	443.407	475.433489	487.398	C_22_H_18_O_10_
	Epigallochatechin gallate	7.308	458.37	459.407	491.433489	503.398	C_22_H_18_O_11_
	Caffeine	8.458	194.19	195.087	227.113489	239.078	C_₈_H_₁₀_N_₄_O_₂_

Green tea root bark	Assamsaponin C	12.214	1231.3	1232.31	1264.333489	1276.3	C_59_H_90_O_27_
	Epichatechin	8.255	290.26	291.277	323.303489	335.268	C_15_H_14_O_6_
	Theobromine	2.774	180.16	181.167	213.193489	225.158	C_7_H_8_N_4_O_2_
	Oxalic acid	2.336	90.03	91.0373	123.063489	135.028	C_₂_H_₂_O_₄_
	Tartaric acid	2.233	150.09	151.097	183.123489	195.088	C_4_H_6_O_6_

^∗∗∗^There were no saponins eluted in the mature green tea leaf extracts.

**Figure 5:**
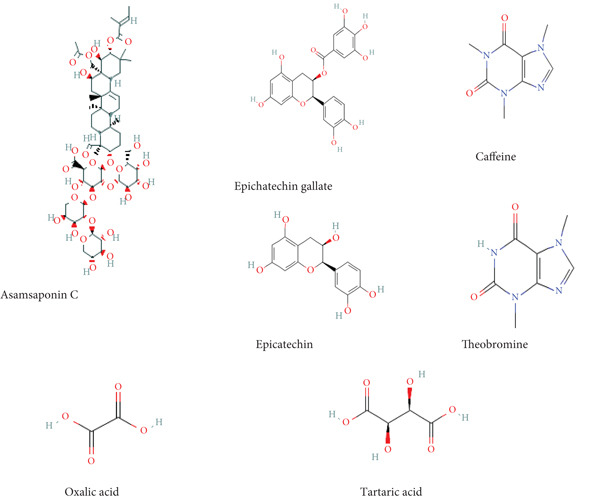
The compound structures retrieved from the National Center for Biotechnology Information (NCBI): PubChem compound summary for Assamsaponin C, epicatechin gallate, caffeine, epicatechin, theobromine, oxalic acid, and tartaric acid.

As shown in Table 11, Assamsaponin C was present in three extracts: green tea seed, purple tea seed, and green tea root‐bark extracts. However, it was not present in the stem bark extracts and the leaf extracts. The extracted ion chromatogram of Assamsaponin C is shown in the Figure S1. Figure 5 shows the chemical structures of the compounds identified by LCMS.

### 3.6. Gas Chromatography

Mass spectra of the eluted compounds were compared against the NIST 17 Library for potential identification. The eluted compounds are shown in Table 12.

**Table 12:** The table below shows the compounds identified by GCMS within the different plants.

**Plant part(s)**	**Compound(s)**	**Retention time**	**Molecular weight**	**Molecular formula**	**Area**	**Area %**
Green tea seed	Caffeine	11.4	194.08	C_8_H_10_N_4_O_2_	1,900,731	55
	Hexadecanoic acid, methyl ester	12.329	270.4507	C_17_H_34_O_2_	273,486	7.91
	9,12‐Octadecadienoic acid (Z,Z)‐, methyl ester	15.091	294.4721		295,138	8.54
	9‐Octadecenoic acid, methyl ester, (E)‐	15.213	296.4879	C_19_H_36_O_2_	503,191	14.56

Purple tea seed	Caffeine	11.406	194.08	C_8_H_10_N_4_O_2_	2,111,689	73.03
	Theobromine	11.644	180.16	C_7_H_8_N_4_O_2_	43,610	1.51
	Hexadecanoic acid, methyl ester	12.334	270.4507	C_17_H_34_O_2_	84,359	2.92
	9,12‐Octadecadienoic acid (Z,Z)‐, methyl ester	15.097	294.4721	C_19_H_34_O_2_	42,765	1.48
	9‐Octadecenoic acid, methyl ester, (E)‐	15.219	296.4879	C_19_H_36_O_2_	214,967	7.43
	9‐Octadecenamide, (Z)‐	20.407	281.4766	C_18_H_35_NO	102,003	3.53

Leaf	Caffeine	11.413	194.08	C_8_H_10_N_4_O_2_	3,094,813	93.47
	Hexadecanoic acid, methyl ester	12.337	270.4507	C_17_H_34_O_2_	38,184	1.15

Stem bark	Beta‐D‐glucopyranoside, methyl	8.291	194.18	C_7_H_14_O_6_	43,632	1.53
	Caffeine	11.408	194.08	C_8_H_10_N_4_O_2_	2,355,113	82.84
	Theobromine	11.651	180.16	C_7_H_8_N_4_O_2_	90,457	3.18
	Hexadecanoic acid, methyl ester	12.335	270.4507	C_17_H_34_O_2_	128,028	4.5
	9,12‐Octadecadienoic acid, methyl ester	15.1	294.4721	C_19_H_34_O_2_	40,682	1.43
	8,11,14‐Docosatrienoic acid, methyl ester	15.215	348.6	C_23_H_40_O_2_	46,575	1.64

Root	Octadec‐1‐ene	8.585	252.4784	C_18_H_36_	28,670	7.25
	Octadec‐1‐ene	10.629	252.4784	C_18_H_36_	26,597	6.73
	Caffeine	11.395	194.08	C_8_H_10_N_4_O_2_	139,088	35.18
	Hexadecanoic acid, methyl ester	12.332	270.4507	C_17_H_34_O_2_	21,525	5.44

In Table 12, the most abundant compound in all the extracts was caffeine. Hexadecanoic acid was also consistently present in all the extracts. The chemical structures of the compounds identified by GCMS are shown in Figure 5.

## 4. Discussion

Synthetic compounds show high mortality rates when used to control snail populations. Despite this efficacy in reducing snail populations, the downside is that their inherent toxicity affects other flora and fauna, including nontarget organisms like fish. Further, Zheng et al. [39] indicate there are other documented side effects that include carcinogenesis, mutagenicity, and teratogenicity. Alternative medicines and natural products show that stages of schistosomiasis can be interfered with through the application of natural products. For instance, Azevedo et al. [40] note that various plant‐derived compounds can have prophylactic activity on adult worms as well as the larval stages of the parasite. Many compounds derived from plants show both antischistosomal and molluscicidal activities at varying levels. Of significance is that these plant‐derived compounds exhibit significantly reduced environmental impacts compared to synthetic compounds [41].

**Figure 6:**
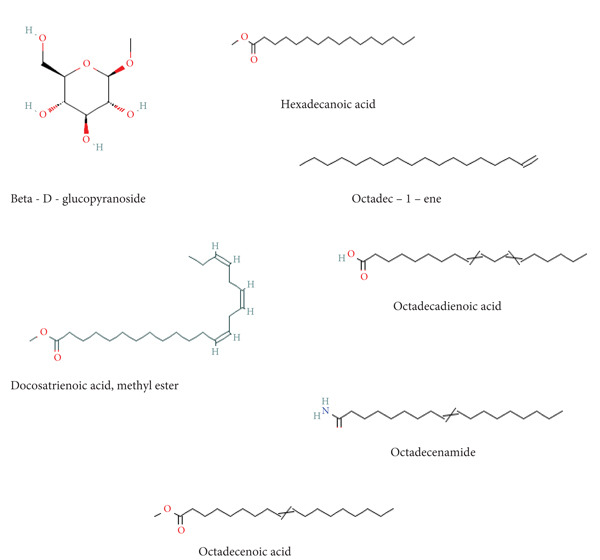
Compounds structures retrieved from NCBI. PubChem compound summary.

In this study, the toxicity assays demonstrated that extracts from green tea and purple tea induced significant retraction and mortality in *Biomphalaria pfeifferi* snails. Notably, snail mortality was most predictable when most snails exhibited retraction within the first 4 h of exposure. Based on the observed mortality rates, the extracts were ranked in descending order of toxicity: purple tea seeds, green tea seeds, root bark, green tea stem bark, leaf shoots, and mature leaves. The likelihood of snail survival was lowest with the seed and root extracts, whereas snails exposed to the leaf extracts exhibited higher survival rates. Furthermore, the probability of survival diminished as the concentration of the extracts increased, indicating an inverse relationship (Figure 4). Phytochemical analysis revealed slight variations in the chemical composition of the different plant parts. Liquid chromatography identified Assam‐saponin C in the green and purple tea seeds, as well as in the green root bark extract, while it was absent in the mature leaves and stem bark (Table 11). The compound structures from LC‐MS are shown in Figure 5. Gas chromatography detected caffeine in all five analyzed extracts, establishing it as the predominant phytochemical across the samples. The GC‐MS compound structures are shown in Figure 6. Additionally, hexadecanoic acid and octadecanoic acid methyl ester were consistently present in the majority of the extracts.

**Figure 7:**
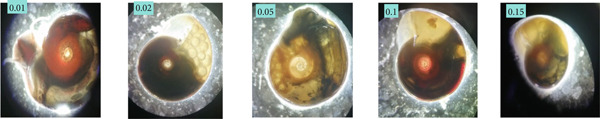
Images of *Biomphalaria pfeifferi* snails under a dissecting microscope (x10 magnification). In the 10‐ppm (0.01) concentration, the snail was visibly intact, and the head was also visibly intact with the antennae. The snail in the 20‐ppm (0.02) concentration was visibly retracted in the shell. In the next concentration of 50 ppm (0.05), the snail body was visibly disintegrating and detaching from the shell. The snail in 100 ppm (0.1) has redness around the snail, which indicates bleeding in the water after exposure. The snail in 150 ppm (0.15) shows the snail with visible body‐mass disintegration around the shell. All the snails shown were exposed to green tea seed extracts, and the images were observed 48 h postexposure.

**Figure 8:**
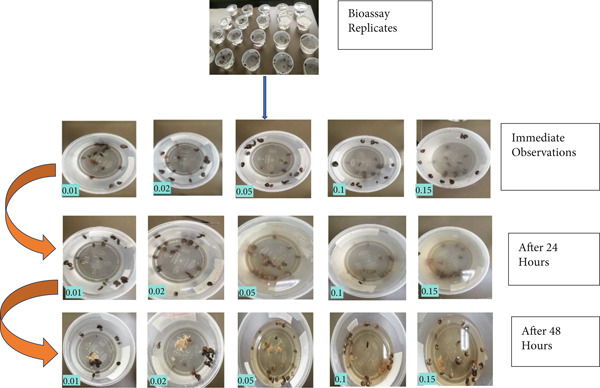
The observations made across the different concentrations at the 24‐h time point and the 48‐h time point.

From our observations, there was visible disintegration of the snail tissues within the test extracts as shown in Figure 7 First, due to the toxicity of the extracts, the snails retracted into their shells, and some moved out of the dishes due to probable irritation. Secondly, 24 h postexposure, the snail body masses detached from the shells, leaving empty shells Figure 8. Some phytochemicals like saponins, which we identified, can induce such toxic effects. From related literature, saponins have been identified as phytochemicals that have potent molluscicidal properties. For instance, Ibrahim et al. [13] tested the effects of saponins on *Bulinus truncatus* snails. The saponins induced tissue damage and other morphological changes within the snails. These changes induced irregular membranes and changes within the hemocytes. Other damages occurred within the cell organelles, like the vacuoles and granulocytes. There was increased secretion of lipid peroxidase, superoxide dismutase, and nitrogen oxide. Increased enzymatic activities were also observed by Akinpelu et al. [11] when investigating the effects of saponins derived from *Erythrophleum suaveolens* against the snail *Lanistes lybicus*. The saponins induced elevated levels of acid as well as alkaline phosphatases. Bahgat et al. [42] highlight that saponins induce apoptosis of some organelles within the snail *Biomphalaria alexandrina*. Other observable effects include increased amoebocytes. Deterioration was observed within the cytoplasm and nucleus. The chromatin within the nucleus was condensed, and there was visible disintegration of the endoplasmic reticulum.

Assam‐saponin C, which we isolated in our extracts, is an organic compound classified as a triterpene saponin. Triterpenoid saponins are plant derivatives found in various plant species. Crude saponins have been extracted from the stem bark of *Erythrophleum suavolens, Pulsatilla chinensis, Albizia anthelmintica, Pueraria peduncularis,* and even *Camellia sinensis* [11, 42–45]. Saponins naturally occur in various plant parts, including stems, fruits, flowers, leaves, pericarps, and seeds. However, the content or abundance of saponin levels may not be the same in every plant; this variation is also observed within various parts of the plant. Some plants may have an abundance of these saponins in seeds compared to the rest of the plant [46]. Saponins are currently utilized as biopesticides in the control of mollusks and other target organisms. Furthermore, saponins possess certain chemical properties that make them useful as viable, low‐cost, environmentally friendly biopesticides [47]. Saponins have surfactant properties, which imply the ability to reduce surface tension between water molecules. Because of their surfactant properties, saponins form foam; foaming is one of their characteristic properties.

The tea plant contains a variety of saponin compounds occurring in different tissues. Wei et al. [48] indicated that the main pathways for the synthesis of saponins are the mevalonic acid (MVA) pathway and the methylethritol phosphate (MEP) pathway. Metabolic pathways ultimately result in the expression of different saponins. For instance, isopentenyl pyrophosphate (IPP) and dimethylallyl diphosphate are identified as 5‐unit precursors for several saponins [46]. In some cases, environmental‐related stresses (biotic and abiotic) may influence the synthesis of various plant compounds, including triterpenoid saponins [48]. Understandably, this mechanism is important for defense against pests and other plant pathogens [49]. Also, the distribution of saponins in plants is dependent on the development stage of the plant. In addition, plant compounds alter surrounding soil microbiomes [50]. Cumulative effects of plant compounds could act as toxins against pathogens. Saponins have shown hemolytic activity, and this activity was demonstrated by Cui et al. [51], whereby the saponin fractions destroyed cell membranes. The destruction of the cell membranes is attributed to the formation of complexes involving cholesterol.

It is suggested that the combinatory effect of plant phytochemicals being used as therapeutic agents could be more effective compared to isolated bioactive compounds [52]. Therefore, phytochemicals work better when in combination with other bioactive compounds to produce a desired effect. In this study, it can be deduced that the compound effect of the phytochemicals within the *Camellia sinensis* exhibited good molluscicidal activity. Even so phytochemicals like polyphenols and terpenoids have shown to be potent whether in isolation or in combinations. Also, their mechanism of action is elucidated. The ability to interfere with membrane proteins is their main mode of action [53]. The greater combinatory effect of phytochemicals suggests that such effects can also be replicated when the plant extracts are used in combination with synthetics as well [54].

## 5. Conclusions

This study sought to evaluate the molluscicidal activity of the Kenyan tea variant (*Camellia sinensis*) against the snails that transmit schistosomiasis. In particular, tea seeds and root bark performed exceptionally, causing the highest snail mortality. This molluscicidal activity could potentially be attributable to specific phytochemicals found in these extracts. Saponins have been shown to have molluscicidal activity. Consequently, tea seeds and root extracts had the presence of Assam‐saponin C, which could be linked to the molluscicidal activity. Other phytochemicals like epigallocatechin gallate and caffeic acid could also be important, acting synergistically and increasing the overall observed activity. The findings of this study confirmed that both green and purple tea have potential as sources of molluscicidal agents.

**Nomenclature**
CBRD: Centre for Biotechnology Research and Development
CTMDR: Centre for Tradition Medicine and Drug Research
ESI: electron spray ionization
GCMS: gas chromatography and mass spectrophotometry
KEMRI: Kenya Medical Research Institute
LCMS: liquid chromatography and mass spectrophotometry
NCBI: National Center for Biotechnology Information.
NTD: neglected tropical disease
NPRC: National Phototherapeutic Research Centre
PZQ: praziquantel
SERU: Scientific and Ethics Review Unit
UoN: University of Nairobi

## Ethics Statement

This study received approvals from KEMRI′s Scientific and Ethics Review Unit (SERU) under the reference number SERU 4683.

## Conflicts of Interest

The authors declare no conflicts of interest.

## Author Contributions

Project administration and investigation, N.S.; supervision and methodology, R.N.; conceptualization and supervision, M.M.; validation, I.M.; formal analysis and data curation, C.K.S; supervision and validation, A.M.; and review and editing, M.L.

## Funding

This work was supported by the Kenya Medical Research Institute, 10.13039/501100019734, KEMRI/IRG/166/6.

## Supporting information


**Supporting Information** Additional supporting information can be found online in the Supporting Information section. Supporting information consists of a figure which shows the extracted ion chromatogram (XIC) of Assamsaponin C from the respective plant extracts.

## Data Availability

The data that support the findings of this study are available from the corresponding author upon reasonable request.
