# Supplementary figures and images for: Molluscicidal Activity of Camellia Sinensis (Green Tea) and Camellia sinensis var. Assamica (Purple Tea) Extracts Against Biomphalaria pfeifferi, the Major Vector Snail of Human Schistosomiasis in Sub‐Saharan Africa
Source: J Parasitol Res. 2026 Jan 2;2026:9932058. doi: 10.1155/japr/9932058 (PMC12767031; doi:10.1155/japr/9932058)

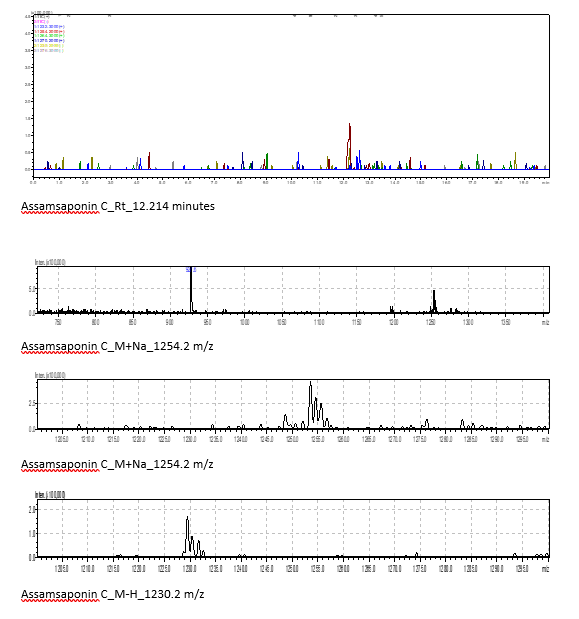

Supplement: Supplementary file 1 — Supporting Information Additional supporting information can be found online in the Supporting Information section. Supporting information consists of a figure which shows the extracted ion chromatogram (XIC) of Assamsaponin C from the respective plant extracts. [file JAPR-2026-9932058-s001.docx]
